# Mutational History of a Human Cell Lineage from Somatic to Induced Pluripotent Stem Cells

**DOI:** 10.1371/journal.pgen.1005932

**Published:** 2016-04-07

**Authors:** Foad J. Rouhani, Serena Nik-Zainal, Arthur Wuster, Yilong Li, Nathalie Conte, Hiroko Koike-Yusa, Natsuhiko Kumasaka, Ludovic Vallier, Kosuke Yusa, Allan Bradley

**Affiliations:** 1 Wellcome Trust Sanger Institute, Hinxton, Cambridge, United Kingdom; 2 Anne McLaren Laboratory for Regenerative Medicine, Department of Surgery, West Forvie Building, Robinson Way, University of Cambridge, Cambridge, United Kingdom; 3 EMBL-EBI, Wellcome Trust Genome Campus, Hinxton, United Kingdom; University of Washington, UNITED STATES

## Abstract

The accuracy of replicating the genetic code is fundamental. DNA repair mechanisms protect the fidelity of the genome ensuring a low error rate between generations. This sustains the similarity of individuals whilst providing a repertoire of variants for evolution. The mutation rate in the human genome has recently been measured to be 50–70 de novo single nucleotide variants (SNVs) between generations. During development mutations accumulate in somatic cells so that an organism is a mosaic. However, variation within a tissue and between tissues has not been analysed. By reprogramming somatic cells into induced pluripotent stem cells (iPSCs), their genomes and the associated mutational history are captured. By sequencing the genomes of polyclonal and monoclonal somatic cells and derived iPSCs we have determined the mutation rates and show how the patterns change from a somatic lineage *in vivo* through to iPSCs. Somatic cells have a mutation rate of 14 SNVs per cell per generation while iPSCs exhibited a ten-fold lower rate. Analyses of mutational signatures suggested that deamination of methylated cytosine may be the major mutagenic source *in vivo*, whilst oxidative DNA damage becomes dominant *in vitro*. Our results provide insights for better understanding of mutational processes and lineage relationships between human somatic cells. Furthermore it provides a foundation for interpretation of elevated mutation rates and patterns in cancer.

## Author Summary

The mutation load of human tissues is unknown and represents the genetic divergence from the fertilised egg. Reprogramming of somatic cells generates induced pluripotent stem cells (iPSCs), a cell type being considered for clinical applications. We generated iPSCs from tissues of healthy individuals and used whole genome sequencing to identify *in vivo* mutations accrued in a somatic cell during the lifetime of the individual. Next we identified *in vitro* mutations introduced during reprogramming and cell culture. Each has a unique mutation signature suggesting different mutagenic processes. Our study demonstrates the use of reprogramming as a tool to elucidate mutational processes within normal cells and highlights the importance of genetic characterisation of iPSCs prior to clinical translation.

## Introduction

From the moment of fertilisation, as each cell divides random mutations occur which are fixed and inherited by daughter cells. Most of these variants have little, if any, physiological consequence but contribute to genetic diversity within tissues. A small proportion will contribute to pathogenic processes such as cancer [1]. Whole genome sequence analysis of cancer genomes has revealed their mutational landscape [1–4]. Cancers are clonally heterogeneous, like the somatic tissues from which they originate, and arise through a series of clonal expansions over decades often acquiring aberrant DNA repair processes [3,5,6]. Thus, the extent to which mutational signatures in human cancers reflect normal non-pathological mutational patterns that have arisen in their normal non-cancerous somatic ancestors is obscure. The mutations that have arisen in somatic cells throughout development and tissue homeostasis are generally difficult to identify in tissue biopsies because these are composed of heterogeneous polyclonal populations of cells.

**Fig 1:**
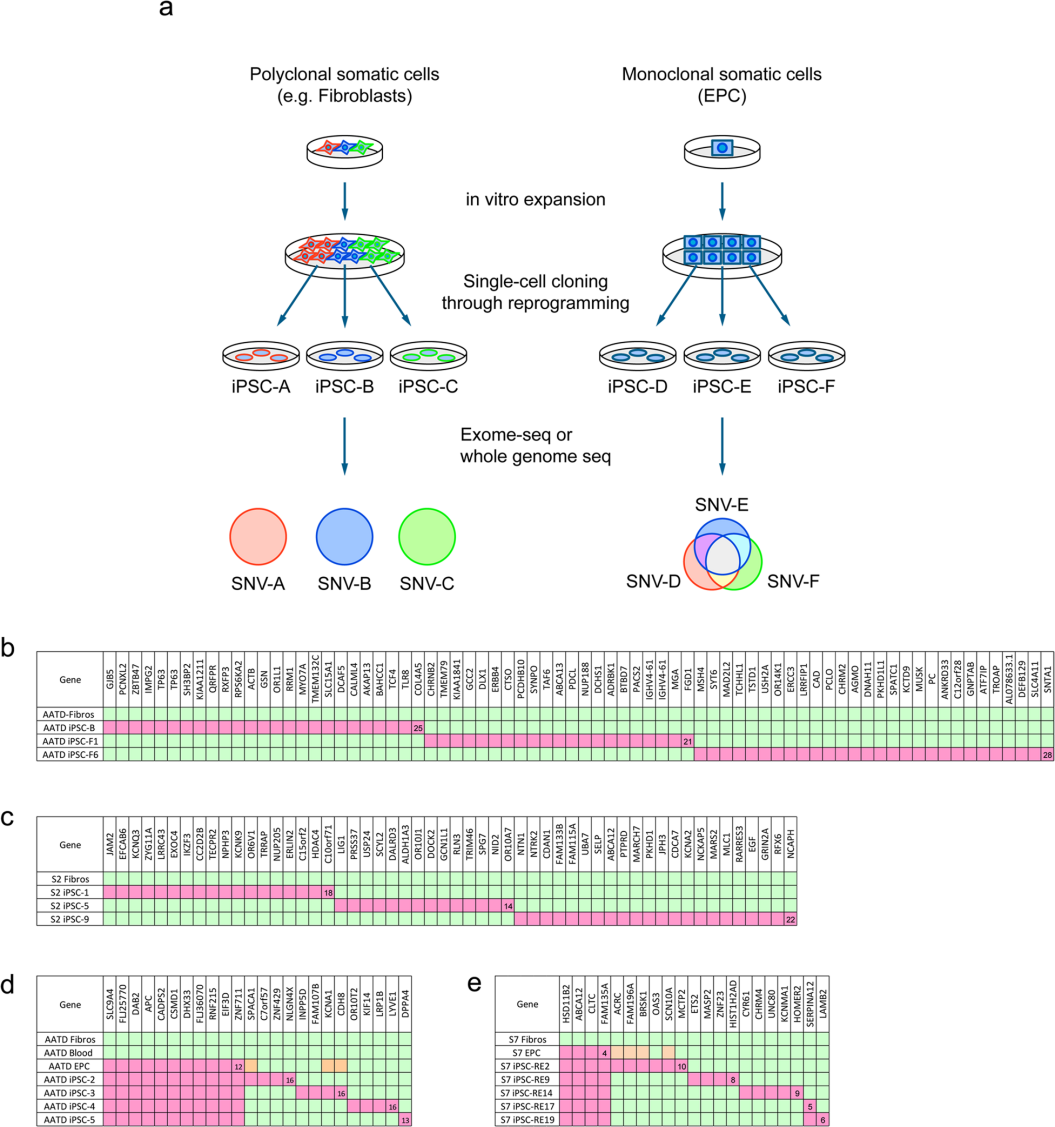
Comparing acquired SNVs in iPSCs derived from a polyclonal or a monoclonal origin. **a**. Schematic comparing the reprogramming of polyclonal and monoclonal cells. Polyclonal cells such as fibroblasts (left panel) give rise to iPSCs which do not share a majority of mutations since they are derived from different progenitors. In contrast, iPSCs derived from monoclonal cells (right panel) such as EPCs share a proportion of their mutations and carry private mutations specific to each line. **b, c**. Exome sequencing of iPSCs generated using fibroblasts from two different individuals, a 65-year-old alpha-1 antitrypsin deficiency patient (AATD) (**b**) and a healthy subject, S2 (**c**). The data for iPSC-B were taken from our previous work [11]. Each column represents one SNV in the indicated gene. Duplicated genes indicate two adjacent SNVs. Green, mutation absent; pink, mutation present. See S2 and S3 Tables for the complete description. **d, e**. Exome sequencing of iPSC lines generated using monoclonal EPCs from the same AATD patient in **b** as well as a healthy subject, S7 (**e**). Orange, mutation detected by amplicon resequencing. See S4 and S5 Tables for the complete description.

To describe the landscape of mutations in normal somatic tissues, we sought to resolve the underlying heterogeneity of somatic tissues by reprograming the constituent cells into induced pluripotent stem cells (iPSCs) [7], a process of single cell cloning that facilitates subsequent expansion. Each clonal iPSC line generated from a heterogeneous polyclonal pool will carry a constellation of mutations reflecting both somatic and culture-induced mutations. Indeed previous work has suggested that a proportion of iPSC mutations originate from the founder somatic cell [8,9]. However although genome sequence analysis of these clones will reveal their mutational burden, it is not possible to definitively resolve the mutations which arose *in vivo* from those which arose during *in vitro* culture and reprogramming (Fig 1A). To confidently classify the origin of the mutations, we derived iPSC lines using monoclonal derived endothelial progenitor cells (EPCs) [10]. The iPSCs isolated from a monoclonal source would share the mutations of the founder cell (*in vivo* acquired somatic mutations) and in addition carry culture-induced mutations as unique private mutations. Sequencing of these iPSCs would allow interrogation of the number and pattern of somatic mutations present *in vivo* (Fig 1A).

## Results and Discussion

Fibroblasts and/or monoclonal EPC lines were derived from three individuals: a 65-year old alpha-1 antitrypsin deficiency male (patient AATD [12]), a 22-year old healthy male (S2 [13]) and a 57-year old healthy male (S7 [13]), which were reprogrammed into iPSCs. The iPSC lines were initially screened using array-based comparative genomic hybridization (CGH) to select lines with the smallest number of copy number aberrations (S1 Table). In addition none of the lines selected had large scale loss of heterozygosity (LOH) through error-prone break recombination (S1 Fig [14]). Next we sequenced the protein-coding exons of these iPSC lines to determine the number and genomic location of their somatic mutations (Fig 1B–1E and S11–S14 Tables). Fibroblast-derived iPSCs from both individuals carried similar numbers of coding mutations, ranging between 14 and 28 single nucleotide variants (SNV) per line (Fig 1B and 1C). Consistent with a polyclonal origin, these SNVs were unique to each line and no shared SNVs were identified between lines from the same individual (Fig 1B and 1C). In contrast, monoclonal EPC-derived iPSC lines (iPSC-2, 3, 4 and 5 from AATD and iPSC-RE2, RE9, RE14, RE17 and RE19 from S7) carried fewer mutations, of which a subset was shared between them as well as with EPCs from the same individual. None of the shared SNVs were detected in the corresponding fibroblasts or whole blood, indicating that these SNVs were somatically acquired by the EPCs *in vivo* (Fig 1D and 1E). In addition, private SNVs were detected which were unique to each monoclonal-derived iPSC line and these were not found in EPCs or the individual’s reference genome. Deep sequencing of the donor EPC genome revealed that some of the mutations detected in the iPSCs were in fact present in the EPCs but at very low frequencies (Fig 1D and 1E, orange boxes; S7 and S8 Tables), suggesting that these mutations were acquired by the EPCs during the *in vitro* expansion process, prior to reprogramming. Notably no known driver mutations (using COSMIC database), which could confer a selective advantage, were identified in any of the iPSC lines. These results demonstrate that iPSCs derived from monoclonal somatic cells can be used to identify *in vivo* acquired somatic mutations.

**Fig 2:**
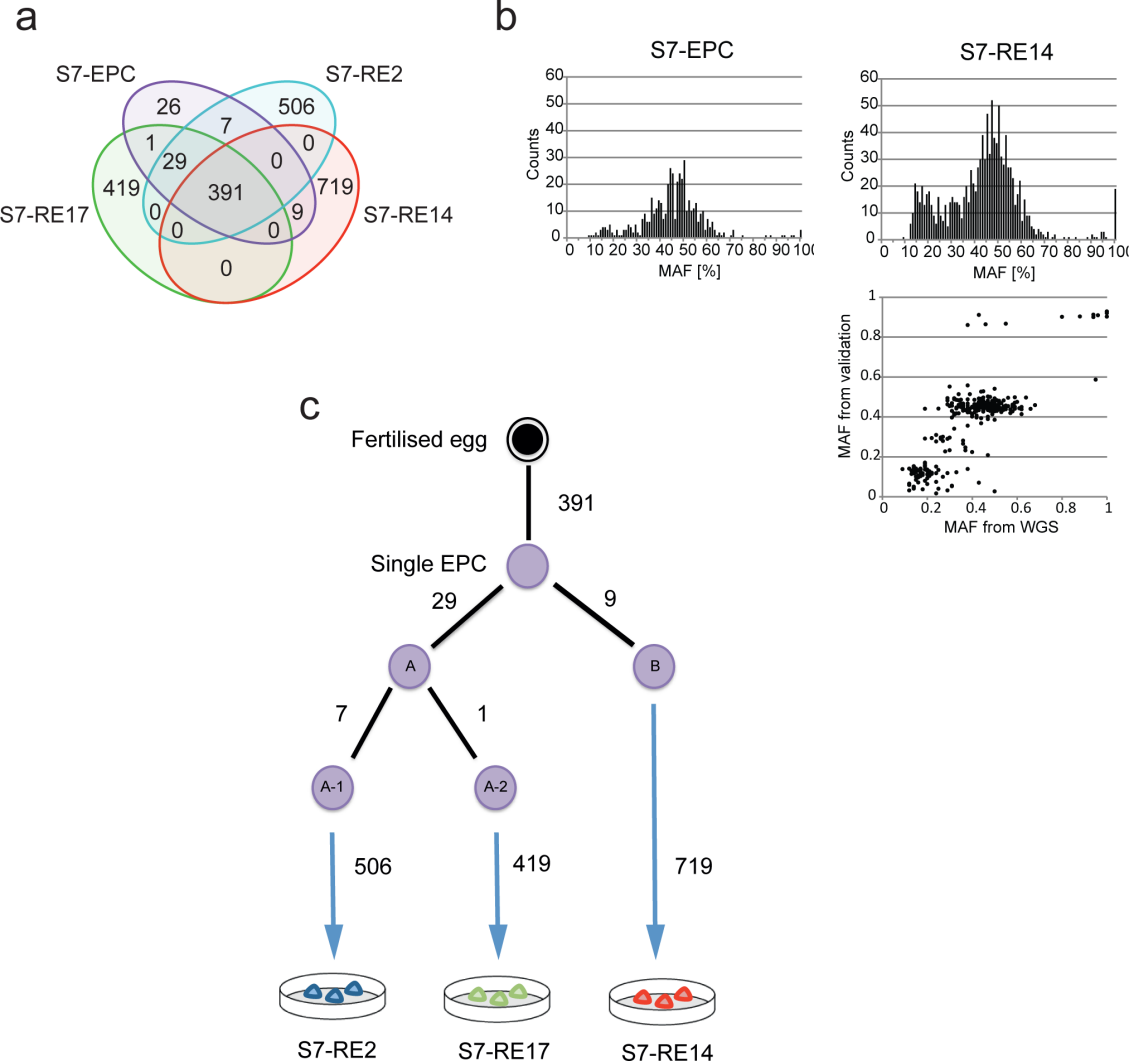
Whole genome sequencing and detailed analysis of iPSCs derived from a monoclonal somatic cell reveal lineage relationships *in vitro*. **a.** Venn diagram showing the overlap of mutations found in each cell line. **b**. Histogram showing mutant allele frequencies (MAF) of SNVs found in EPCs and S7-RE14. Note the presence of sub-clonal SNVs (<30% MAF). MAFs observed from amplicon resequencing revealed 3 distinct sub-populations, which represent clonal SNVs, SNVs fixed during the first and second cell division (bottom panel). **c**. Cellular phylogenetic tree showing the relationship between the first and second cell divisions of the originating EPC and the subsequent iPSC line. The minimum number of mutations (taking into account limitations of the sensitivity of detecting sub-clonal mutations by whole genome sequence) accrued by each daughter cell is shown.

The mutational burden of iPSCs reflects mutations accumulated *in vivo* in the ancestral somatic cell lineages and mutations acquired during *in vitro* cell culture and subsequent reprogramming. The iPSCs from heterogeneous somatic cells usually do not share any mutations but the exome sequencing data demonstrated that by using monoclonal cell sources it is possible to resolve mutations acquired *in vivo* from those arising during *in vitro* cell culture. Furthermore, identifying shared mutations in somatic cell lineages could be used to construct a cellular phylogenetic tree. We therefore performed whole genome sequencing on the S7-derived monoclonal EPCs, 3 iPSC lines (RE2, RE11 and RE14) and fibroblasts, which were used as the reference genome (S9 Table). The total number of mapped bases obtained per sample was 108.1–122.8Gb with 33 – 37X sequence coverage. We identified 463 SNVs in the monoclonal EPCs and 933, 1119 and 840 in the iPSCs, respectively (Fig 2A). A proportion of the putative SNVs were validated using PCR amplicon re-sequencing. This analysis revealed that we were able to detect SNVs with mutant allele frequencies of less than 30% with high specificity (S10 Table), which most likely represent mutations acquired during the first few divisions after founder cells started dividing (Fig 2B).

Amongst the SNVs called, 391 mutations were shared by all the iPSC lines and the monoclonal EPCs at a mutant allele frequency of approximately 50%, which is consistent with clonal mutations (heterozygous SNVs in diploid chromosomes). Therefore these 391 SNVs reflect the *in vivo* genetic divergence of the single EPC from fertilisation through development and adulthood. Some SNVs were shared between the EPCs and only a subset of the lines (Fig 2A), revealing the emergence of genetic differences during *in vitro* EPC culture. The remaining SNVs were unique to each iPSC line and not present in the EPCs at a detectable frequency. These private mutations in RE2 (506 SNVs), RE17 (419 SNVs) and RE14 (719 SNVs) represent *in vitro* SNVs acquired in the EPC culture and/or during reprogramming (S2–S6 Tables).

The SNVs detected in the EPCs and iPSCs are a historical record of the phylogenetic lineage of the cells (Fig 2C). For the individual S7, in the 57 years from fertilization to the point of derivation of the single EPC, 391 mutations had accumulated *in vivo*. The single EPC was then expanded *in vitro* prior to reprogramming. Following the first cell division of the EPC, one daughter cell (A) acquired at least 29 mutations and the other daughter cell (B) at least 9 mutations. After daughter cell A divides, two further branches appear resulting in at least 7 mutations in one granddaughter cell (A-1) and at least 1 mutation in the other (A-2). The progeny of daughter cells A-1, A-2 and B were the eventual substrates for the derived iPSC lines S7-RE2, S7-RE17 and S7-RE14, respectively.

The detailed mutation analysis we performed enabled us to estimate the *in vitro* mutation rate of the EPCs. Apart from the 391 *in vivo* mutations, the clonal SNVs detected in the iPSCs were acquired during the EPC expansion and reprogramming and thus should be present in parental EPCs. We sought to detect these sub-clonal mutations that are present in EPCs by deep sequencing and calculate a mutation rate during *in vitro* EPC expansion using a statistical model (See Materials and Methods). First, in order to ensure accuracy especially at the lower bound of allele frequencies, we investigated sequencing error rates. Eight genomic regions (S15 Table and S2 Fig) were PCR-amplified from the AATD iPSC-B cells and sequenced on a MiSeq instrument. Median error rates were 0.042–0.144% and 0.053–0.320% for the first and second reads respectively when the first and second reads were analysed separately. However, median error rates were substantially improved (0.016–0.025%) when consensus sequences were first generated from the first and second reads and then bases were counted (S2 Fig). We used this approach to accurately identify low-frequency subclonal mutations.

We amplified approximately 40% of the in vitro SNVs from genomic DNA derived from the S7 EPCs and performed deep sequence analysis. Of this subset, we detected 60, 51 and 58 SNVs in S7-RE2, S7-RE14, and S7-RE17 respectively to be present in the EPCs at allele frequencies between 41% and 0.05% (Table 1). The sub-clonal SNVs in the EPCs were then used to calculate the mutation rate during *in vitro* culture, resulting in an estimated mutation rate of 14.0 ± 2.0 SNVs per cell per generation or 2.1 x 10^−9^ per nucleotide per generation (see Materials and Methods).

**Table 1:** Summary of WGS and deep sequence analysis.

iPSC line	SNVs detected	*In vivo* acquired SNVs	*In vitro* acquired SNVs	No. (%) of SNVs analyzed by deep sequencing	SNVs detected in EPC by deep sequencing
**S7 RE2**	933	391	542	228 (42.1)	60
**S7 RE14**	840	391	728	319 (43.8)	51
**S7 RE17**	1119	391	449	167 (37.2)	58

Three iPSC lines derived from EPCs from the same individual were analyzed by WGS. Of the 840–1119 SNVs detected, 391 SNVs were shared by all the lines implying that these were present in the originating EPC, having accumulated *in vivo*. A proportion of the remainder SNVs were analyzed by PCR amplification and deep sequencing in order to detect SNVs that were present in the EPCs at a low frequency. 51–60 SNVs were detected within the EPC population by this method.

**Fig 3:**
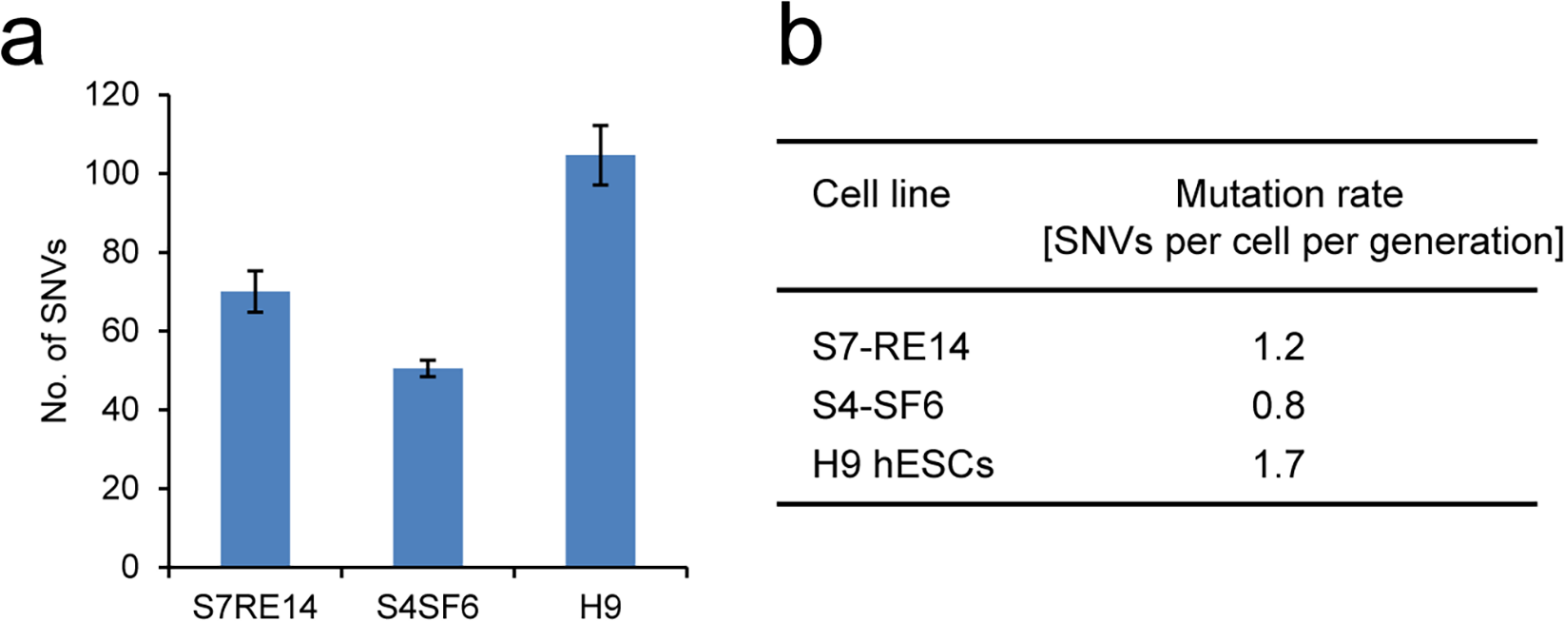
Mutation rate of human pluripotent cells in culture. **a**. The mean numbers of SNVs accumulated during 60 cell divisions in 2 iPSC lines, S7-RE14 (*n* = 3) and S4-SF6 (*n* = 2) and a human ESC line H9 (*n* = 3). Data are shown as mean ± SD. **b**. Mutation rate per cell per division in each pluripotent cell line.

Clinical use of iPSCs requires not only generation but also maintenance of iPSCs in cell culture. We therefore sought to measure the rate of single nucleotide mutagenesis in iPSCs. In order to calculate this precisely, we sub-cloned iPSCs from individuals S7 and S4 (a 61-year old healthy female) as well as H9 human embryonic stem (ES) cells [15] and grew these continuously for 60 divisions. At the end of the expansion period, we sampled the population from each cell line by sequencing single cell sub-clones that had been expanded to provide an adequate DNA sample for whole genome sequencing. Comparison of the DNA sequence from these sub-clones to its immediate parental population identified *in vitro* mutations acquired during 60 divisions. All three lines had a similarly low mutation rate of 0.8–1.7 SNVs per cell per generation or 1.8 x 10^−10^ per nucleotide per generation (Fig 3A and 3B). Intriguingly, although both EPCs and pluripotent stem cells have a similar cell cycle time, the mutation rate in pluripotent stem cells was approximately tenfold lower than that in EPCs during *in vitro* culture.

**Fig 4:**
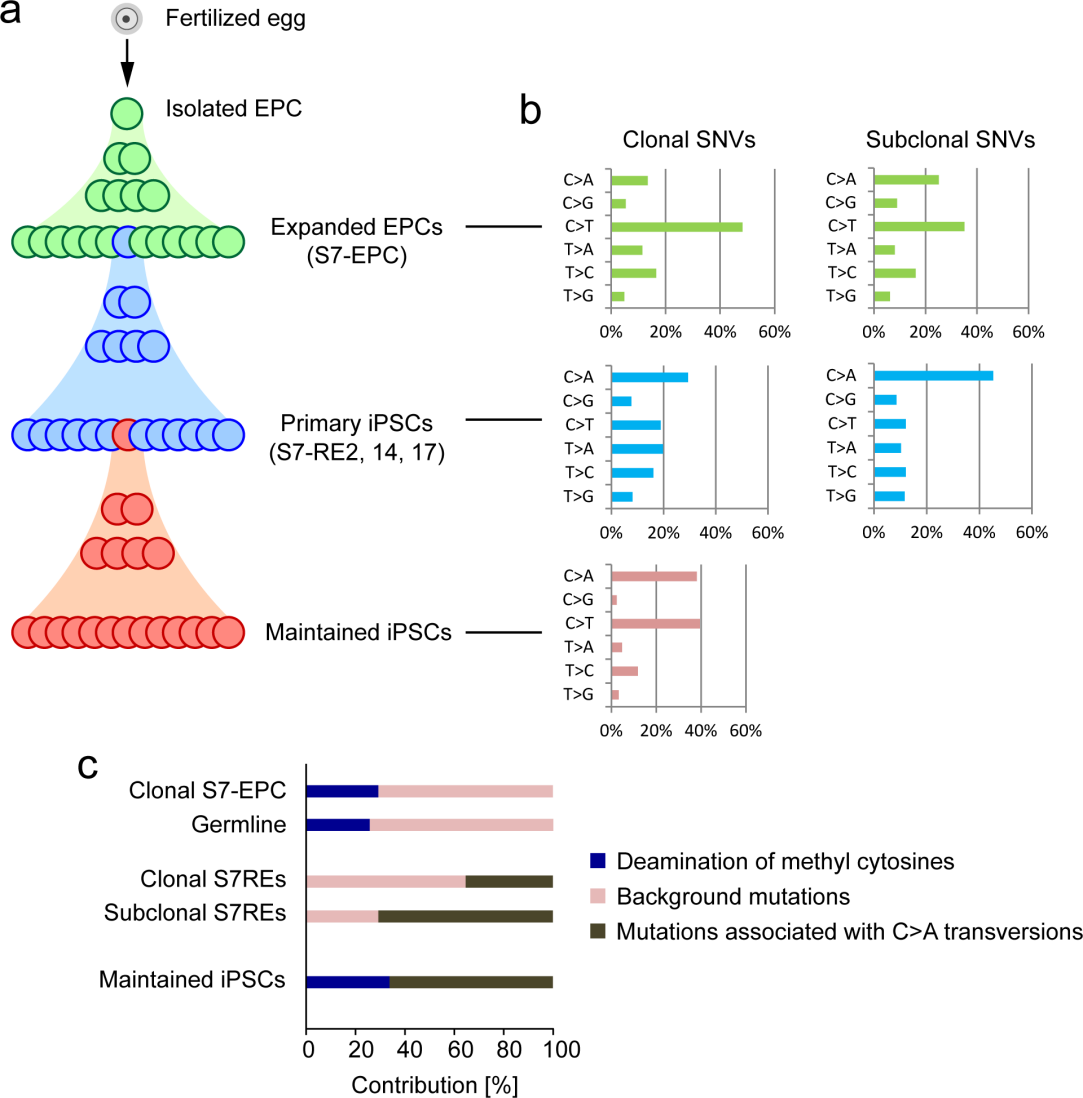
Mutational signatures *in vivo*, *in vitro* and through reprogramming. **a**. Schematic showing the longitudinal progression from *in vivo* development, *in vitro* culture of somatic cells through reprogramming and finally through to the experimental set-up used to calculate the mutation rate in iPSC maintenance culture. **b**. Mutational spectrum of SNVs found in the EPCs (top), and primary (middle) and sub-cloned (bottom) S7-RE14 iPSC lines. Clonal (left) and sub-clonal (right) mutations were shown separately. **c**. Contribution of mutational processes identified by the NNMF analysis. The germ line mutations described in ref 18 were analysed [18]. The NNMF analysis was not performed for the mutations in sub-clonal S7-EPC due to the limited number of mutations available.

Next, we sought to understand whether the patterns of the mutations could inform us of the mutagenic processes involved both *in vivo* and during *in vitro* cell culture. We separated the S7 mutations into three groups that represented the continuous cellular lineage for this 57-year old man, from fertilisation to isolation of the single EPC (*in vivo*), expansion of the EPCs and reprogramming (*in vitro* somatic cells) and finally maintenance of the iPSCs (*in vitro* iPSCs) (Fig 4A). Using a Bayesian Dirichlet process [16,17] we were able to model clusters of clonal and subclonal (generated after the 1^st^ cell division; <30% MAF) SNVs for each cell population. We explored the types of base substitutions seen in these groups of mutations and found variation in the overall mutation spectra (Fig 4B). There is a preponderance of C:G>T:A transitions *in vivo* and early in the cellular lineage. In contrast, *in vitro* and later in the cellular lineage, there is a preponderance of C:G>A:T transversions.

To explore mutational processes in more detail, we conducted Non Negative Matrix Factorization (NNMF) analysis [4]. Firstly, we found that the clonal mutations in S7-EPCs, representing somatic substitutions acquired *in vivo*, are associated with a signature that has been attributed to deamination of methylated cytosines, a process thought to occur in all cells. This signature is similar to the mutations observed in germ cells, another example of *in vivo* mutations in normal cells (Fig 4C). Secondly, the mutation signatures acquired by the EPC population *in vitro* (clonal S7REs) were composed of a combination of deamination and C>A transversions. We speculate that this latterly acquired signature represents damage accrued during culture and may be due to oxidative DNA damage [19]. Thirdly, we detected a sharp increase in the proportion of mutations associated with C>A transversions in sub-clonal mutations in the iPSCs (subclonal S7REs). These sub-clonal mutations detected in iPSCs arise in the first few cell cycles after a clonal cell line appears. Cells during this period are thought to be undergoing reprogramming, suggesting that iPSC reprogramming may stimulate a mutational process associated with C>A transversions. Finally, the *in vitro* mutations of iPSCs (maintenance cell culture) were associated with both deamination of methylated cytosines and the C>A transversions, reinforcing the suggestion that it is a putative imprint of culture-related/oxidative damage *in vitro*.

We have extensively analysed a series of normal single-cell derived clones by whole genome and exome sequencing. We report for the first time the number and characteristics of the acquired mutations in a monoclonal cell isolated from a healthy individual and subsequently derived iPSCs. From this data we are able to reconstruct the mutational history of a cell beginning from the fertilised egg through to adulthood, then to reprogramming and maintenance of iPSCs in long-term culture, demonstrating how mutagenic processes evolve through that cellular lineage. During first *in vivo* then *in vitro* cell divisions, there is a change in the mutation signatures, suggesting a proportional reduction in the contribution of deamination of methylated cytosines and a proportional increase in oxidative stress and DNA damage. Finally, consistent with the expectation that an organism should protect its stem cells, we observed a ten-fold reduction in mutation rate in iPSCs, which mirrored that in human ES cells, which have not been subjected to reprogramming.

We find that reprogramming is mutagenic at the nucleotide level and, similar to previous reports [20,21], not at the chromosomal level. The nucleotide-level mutations are associated with a sharp increase in the proportion of mutations associated with oxidative DNA damage. However established iPSCs seem to be substantially protected from DNA damage by their pluripotent state. The increased DNA replication fidelity of iPSCs and ES cells may be due to the activity of homologous recombination throughout the cell cycle in pluripotent cells, whereas in somatic cells it is restricted to the stages of the cell cycle in which there is presence of replicated chromatin [22,23]. Although *in vitro* culture of iPSCs has a reassuringly low mutation rate, the culture systems used altered the mutational spectrum, which shifted from predominantly C>T transitions to C>A transversions. Over the relatively few generations we studied, we could not find any evidence of a selection sweep within the culture. Notably we did not find any driver mutations in our analyses. Understanding how mutations accrue through iPSC reprogramming and during maintenance cell culture is paramount to developing safe clinical therapies. Furthermore the mutational signatures underlying normal development and tissue homeostasis provide insights into the biological processes occurring in normal cells.

## Materials and Methods

### Procurement of tissues

Primary tissue samples and blood were obtained from a patient with alpha-1 antitrypsin deficiency (patient 2) under the ethics approval REC No. 08/H0311/201 or adult cadaveric organ transplant donors referred to the Eastern Organ Donation Services Team (part of NHS Blood and Transplant). Ethics approval for the latter was obtained from Cambridgeshire Research Ethics Committee 3 (REC No. 09/H306/73). All laboratory procedures were performed according to Standard Operating Protocols and safety assessments.

### Derivation of fibroblasts

For each subject included in this study, around 3cm of skin was excised from the midline surgical incision. The fat and dermal layers of the skin sample were removed and the skin was cut into approximately 1mm^3^ pieces. These were dispersed evenly on a 10cm plate (maximum 20 pieces) and incubated with fibroblast growth media (Knockout DMEM with 20% FBS). At 21 days the fibroblasts were harvested using trypsin.

### Derivation of endothelial progenitor cells (EPCs)

For each derivation, 100mL of blood was taken from the patient into two 50mL Falcon tubes each containing 5mL of 10% sodium citrate. The sample was mixed by inversion and transporting to the laboratory on ice. The blood samples were diluted 1:1 with Ca^2+^ and Mg^2+^ free PBS and 20mL was layered gently onto 15mL of Ficoll Paque Plus (GE Healthcare) and centrifuged at 400g for 35min. The buffy coat containing the mononuclear cells was transferred into a new Falcon tube, diluted 1:1 with PBS and the cells were pelleted by centrifugation at 300g for 20min. Cell pellets were re-suspended in 15mL of EPC media: EGM-2MV supplemented with growth factors (Lonza) supplemented with 20% FCS (HyClone), and plated onto collagen coated T-75ml flasks (BD Biosciences) [10]. The media was changed every 2 days and colonies started appearing from Day 10. After 21 days the EPCs were passaged using trypsin and re-plated into a new T-75 flask (without collagen). The cells were expanded through sequential passages in 1:3 ratios.

### Culture of human iPSCs and ES cells

H9 hESCs were obtained from WiCell Research Institute. Human iPSCs and ES cells were maintained as described previously [11,15]. Briefly, the cells were cultured on irradiated mouse embryonic fibroblast (MEF) feeder layers in iPSC medium (termed KSR + FGF-2): Advanced DMEM/F12 (Invitrogen) supplemented with 20% Knockout Serum Replacement (Invitrogen), 2mM L-glutamine (Invitrogen), 0.1mM β-mercaptoethanol (Sigma-Aldrich) and 4ng/mL of recombinant human basic Fibroblast Growth Factor-2 (R&D systems). Medium was changed daily and the cells were passaged every 5–10 days depending on the confluence of the plates. To split iPSCs and ES cells, the plates were washed in PBS and 3mL of each of collagenase and dispase was added (Collagenase IV 1mg/mL, Invitrogen; Dispase 1mg/mL, Invitrogen).

### Reprogramming

For retroviral reprogramming, four pseudo-typed Moloney murine leukaemia retroviruses containing the coding sequences of each of human POU5F1, SOX2, KLF4 and MYC were obtained from Vectalys. For each iPSC derivation, 1 x 10^5^ primary cells (fibroblasts or EPCs) were plated one day before transduction. The 4 viruses were added at a multiplicity of infection of 10 along with 10 μg/mL of polybrene (Millipore). The following day residual virus was washed off with PBS and the cells were re-fed with the fresh medium. On day 5 after infection, the cells were re-plated using trypsin onto a 10cm dish of fresh MEF feeders and 2 days later, the medium was changed from primary cell-specific media to the iPSC medium (KSR + FGF-2). The medium was changed every 2 days until colonies emerged after which the medium was changed daily. For Sendai virus-mediated reprogramming, four viruses containing the coding sequences of human POU5F1, SOX2, KLF4 and MYC were obtained from DNAVec. The protocol for reprogramming was identical to that of retroviruses except that 5 x 10^5^ fibroblasts were used at a multiplicity of infection of three and polybrene was omitted.

The iPSC colonies were identified by their morphology and picked once they had reached sufficient size, typically from day 25 following transduction. Each colony was first detached from the surrounding feeders by scoring around the circumference. The colony was then split into quarters or eighths and the segments gently lifted off the plate and transferred to one well of a 12 well plate of fresh MEF feeders containing iPSC media (KSR + FGF2) supplemented with ROCK inhibitor (Y-27632, Sigma) [24]. The majority of the iPSCs used in this study have been previously characterised in other publications [12,13].

### Array-based comparative genomic hybridization and exome sequencing

This was performed as described previously [11].

### Whole genome sequencing library preparation and alignment

Genomic DNA was extracted from cell pellets using the DNeasy Blood and Tissue kit (Qiagen). Short-insert 500bp whole genome libraries were constructed, flowcells prepared and sequencing clusters generated according to the manufacturer’s protocols and sequenced using the Illumina HiSeq2000 platform (100bp paired-end). Short-insert paired-end reads were aligned to the reference human genome (GRCh37/hg19) using the Burrows-Wheeler Aligner (BWA) [25], duplicates removed. The average sequence coverage was 34-fold.

### Calling of somatic variants

Somatic base substitution mutations were called using CaVEMan (Cancer Variants Through Expectation Maximization: http://cancerit.github.io/CaVEMan/) which provides a probabilistic estimate of a variant being a somatic mutation. Only variants with likelihoods of 95% and above were included. Post-hoc filters (previously trained on 21 WGS cancers [3]) that sought to remove systematic sequencing artifacts as well as artifacts that arise from mapping errors, were applied to reduce the false positive rate.

### SNV validation

SNVs, for which PCR primers could be designed, were all analyzed by amplicon re-sequencing. PCR primers were designed using BatchPrimer3 to amplify regions spanning SNVs. PCR was performed with 5ng of genomic DNA (Fibroblasts, EPCs and iPSCs) used as a template with Phusion Hot Start DNA Polymerase with GC buffer in the following conditions: 98°C for 1 min, 35 cycles of 98°C for 15 sec, 58°C for 15 sec and 72°C for 30 sec, followed by the final extension, 72°C for 5 min. PCR products were first pooled by sample and then purified with QIAquick PCR Purification Kit (Qiagen). Purified PCR products from A1ATD patient B-derived EPCs were converted to a 454 library by emulsion-PCR and sequenced using the 454 Titanium platform according to the manufacturer’s instruction. Purified PCR products from the other samples were converted to an Illumina library by adaptor ligation and sequenced on either the MiSeq (150bp, paired end) or the HiSeq2000 (100bp, paired end) platforms. Reads from the 454 platform were aligned to a reference constructed from PCR-amplified regions. Paired end reads from the MiSeq or HiSeq2000 were first used to generate consensus sequences between each pair and then these were aligned to a reference using BWA SW [25]. The number of reads reporting each of the four bases was counted using Samtool.

### Detection of subclonal mutations in EPCs

PCR primers were designed in a way that each SNV was located in a region where both Illumina reads could reach. PCR and Illumina sequencing were performed as described above. Fastq files (1.fq and 2.fq) were first merged to generate consensus sequence reads. In this process, base calls were accepted only when a sum of Q scores from both reads was higher than 40 and both reads reported the same base. Reads were discarded if an overlapping region exhibited more than 10% mismatches between the two reads. Consensus reads were subsequently mapped onto the reference sequence using BWA SW and the number of reads reporting each of the four bases was counted using Samtool. Two-way contingency Chi-square tests were performed between the reads reporting reference and mutant variants and between fibroblasts and EPCs. Multiple test correction was performed using the Bonferroni correction. SNVs whose mutant read was significantly higher in EPCs were counted as subclonal mutations. Analyses on the subclonal SNVs with less than 0.1% were shown in S16 Table.

### Estimation of SNV mutation rate in EPCs

It is not possible to subclone and serially expand EPCs therefore a statistical model was used to estimate the SNV mutation rate in EPCs. We obtained 13.5 x 10^6^ cells at the end of S7-EPC expansion, which represents that a single EPC underwent approximately 24 cell divisions. When 5ng (approximately 750 cells or 1,500 molecules) were used as a template for each PCR, assuming that the sampling of DNA molecule follows the Poisson distribution, probability of sampling *k* number of DNA molecules carrying each SNV introduced at generation *n* is therefore given by

$$P_{n}(X=k)=\frac{\lambda_{n}^{k}exp(-\lambda_{n})}{k!},$$

where *λ*_*n*_ (= 1500/2^n+1^) represents the mean molecule number of each mutation introduced at generation *n* in the 5ng DNA. The total number of mutations that can be detected with amplicon re-sequencing is

$$\sum_{n=0}^{24}P_{n}(X>0)M_{ave}=9.88M_{ave},$$

where *M*_*ave*_ is the average mutation rate, assuming that the mutation rate is similar throughout EPC culture. Taking into account the numbers of sub-clonal EPC mutations detected (SNVs detected in EPCs by deep sequencing; Table 1) and the 40% sampling for deep sequence analysis, we estimated mutation rate of 14.0 ± 2.0 SNVs per cell per generation or 2.1 x 10^−9^ per nucleotide per generation.

### Ethics statement

All work performed as part of this project was approved by an ethics committee under the REC Nos. 09/H306/73 and 08/H0311/201.

### Accession numbers

The aCGH data has been deposited with the ArrayExpress under the accession number, E-MTAB-1319. Whole genome sequence data have been deposited with the European Genome-phenome Archive under the accession number EGAS00001000231 and exome data under the accession number EGAS00001000492.

## Supporting Information

S1 Fig Copy number analysis for the iPSC lines. Representative copy number profiles derived from ASCAT [14] for S7 iPSC-RE2 and S7 iPSC-RE14 are shown. Chromosomes are provided on the horizontal axis and integer copy number values are provided on the vertical axis for each clone. Purple lines denote total copy number whilst blue line denotes minor copy number values. All the clones were diploid.
(TIF)

S2 Fig Improvement of sequencing accuracy using consensus reads from AATD iPSC-B. **a**,**b**. Error rates within the region where the first and second reads overlap for Region 1 (**a**) and 2 (**b**). Note that the merged reads consistently showed lower error rates than the first and second reads. **c-e**, Box plots showing error rates in Region 1 (**c**), 2(**d**) and 3–8 (**e**). 95th, 75th, 25th and 5th percentile and the median value are shown. The Mann–Whitney *U* test was performed.
(TIF)

S1 Table Array-CGH analysis of human iPSC lines generated. The a-CGH results of iPSC lines are shown for each donor, indicating the name of the line, donor cell of origin, the genomic abnormality, chromosomal location, size of abnormality and genes affected.
(XLSX)

S2 Table A list of exonic mutations found in iPSC lines derived from fibroblasts of AATD patient. The exome sequencing data for each iPSC line is shown together with the Mutant Allele Frequency (MAF) and consequence of the mutation using SIFT prediction. Each iPSC line is compared to the patient’s fibroblasts, taken to be the reference genome for the AATD patient. Mutations that are present are shown in pink and those that are absent in green.
(XLSX)

S3 Table A list of exonic mutations found in iPSC lines derived from fibroblasts of donor S2. The exome sequencing data for each iPSC line is shown together with the Mutant Allele Frequency (MAF) and consequence of the mutation using SIFT prediction. Each iPSC line is compared to the subject’s fibroblasts, taken to be the reference genome for the S2 donor. Mutations that are present are shown in pink and those that are absent in green.
(XLSX)

S4 Table A list of exonic mutations found in iPSC lines derived from EPCs of AATD patient. The exome sequencing data for each iPSC line is shown together with the Mutant Allele Frequency (MAF) and consequence of the mutation using SIFT prediction. Each iPSC line is compared to the subject’s fibroblasts, taken to be the reference genome for the AATD patient. Mutations that are present are shown in pink and those that are absent in green. Deep sequencing revealed the presence of some of these mutations at low frequency in the EPC population (orange).
(XLSX)

S5 Table A list of exonic mutations found in iPSC lines derived from EPCs of donor S7. The exome sequencing data for each iPSC line is shown together with the Mutant Allele Frequency (MAF) and consequence of the mutation using SIFT prediction. Each iPSC line is compared to the subject’s fibroblasts, taken to be the reference genome for the S7 donor. Mutations that are present are shown in pink and those that are absent in green. Deep sequencing revealed the presence of some of these mutations at low frequency in the EPC population (orange).
(XLSX)

S6 Table A summary of exome sequencing. The number of mapped reads and mean coverage is shown for all exome sequencing experiments.
(XLSX)

S7 Table Deep sequencing analysis of SNVs in AATD patient derived EPCs. Some of the iPSC SNVs were detectable in the EPCs at low frequencies using deep sequencing. The patient’s fibroblasts were used as the reference genome and the p-value for detecting alternative (mutant) alleles is shown.
(XLSX)

S8 Table Deep sequencing analysis of SNVs in S7 donor derived EPCs. Some of the iPSC SNVs were detectable in the EPCs at low frequencies using deep sequencing. The person’s fibroblasts were used as the reference genome and the p-value for detecting alternative (mutant) alleles is shown.
(XLSX)

S9 Table A summary of whole genome sequencing. The read count, mapping and coverage statistics are shown for all WGS data generated for all experiments.
(XLSX)

S10 Table Summary of validation of SNVs for S7 EPCs and iPSCs. The SNVs identified through WGS of S7 EPCs and iPSCs were divided according to Mutant Allele Frequency (MAF) of > 30% (clonal mutations) and ≤ 30% (subclonal mutations). These SNVs were validated using an orthogonal platform in duplicates with high specificity as demonstrated by the low percentage of false positives.
(XLSX)

S11 Table List of shared SNVs detected in S7 EPCs and iPSC lines S7RE2, S7RE14 and S7RE17 including those mutations validated through deep sequencing. The shared SNVs are highlighted in pink as well as those which were validated, in green.
(XLSX)

S12 Table List of SNVs detected in iPSC line S7RE2. For each SNV, the number of mutant reads and depth of coverage compared to controls is shown as well as the mutant allele frequency.
(XLSX)

S13 Table List of SNVs detected in iPSC line S7RE14. For each SNV, the number of mutant reads and depth of coverage compared to controls is shown as well as the mutant allele frequency.
(XLSX)

S14 Table List of SNVs detected in iPSC line S7RE17. For each SNV, the number of mutant reads and depth of coverage compared to controls is shown as well as the mutant allele frequency.
(XLSX)

S15 Table The primer sequences used to amplify 8 AATD iPSC-B genomic sites in order to accurately identify low frequency sub-clonal mutations. The genomic sites relate to the plots in S1 Fig.
(XLSX)

S16 Table The 8 genomic sites where the mutant allele frequencies in S7 EPCs and fibroblasts were <0.1%. At each genomic position, the reference and the mutant bases are shown as well as the mutant allele frequencies in the EPC and fibroblast genomes. The background error rate, the mutant allele frequency in the reference genome (fibroblasts) is approximately 0.01%.
(XLSX)
