# Supplementary figures and images for: Mutational History of a Human Cell Lineage from Somatic to Induced Pluripotent Stem Cells
Source: PLoS Genet. 2016 Apr 7;12(4):e1005932. doi: 10.1371/journal.pgen.1005932 (PMC4824386; doi:10.1371/journal.pgen.1005932)

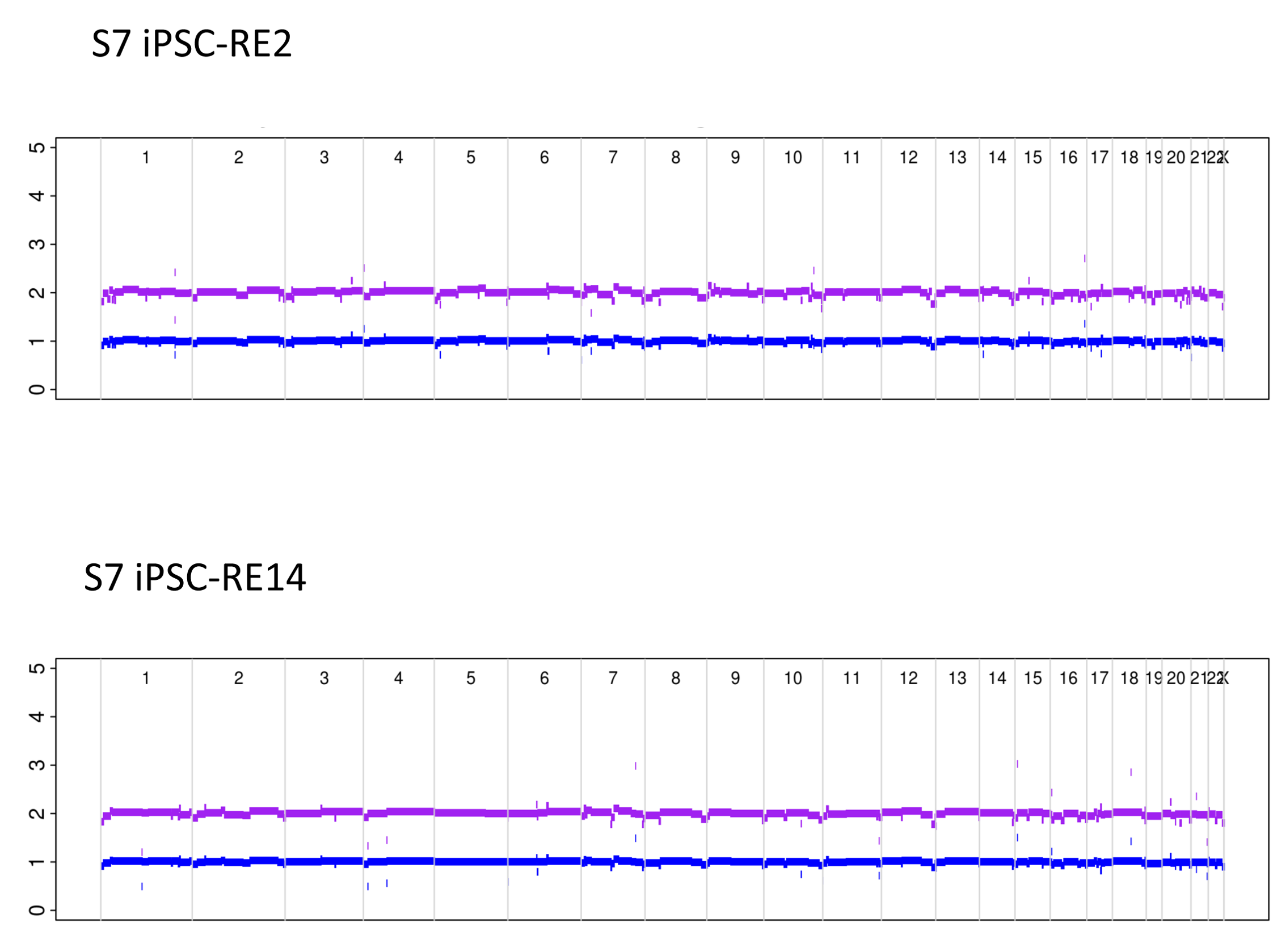

Supplement: S1 Fig — Representative copy number profiles derived from ASCAT [14] for S7 iPSC-RE2 and S7 iPSC-RE14 are shown. Chromosomes are provided on the horizontal axis and integer copy number values are provided on the vertical axis for each clone. Purple lines denote total copy number whilst blue line denotes minor copy number values. All the clones were diploid. (TIF) [file pgen.1005932.s001.tif]

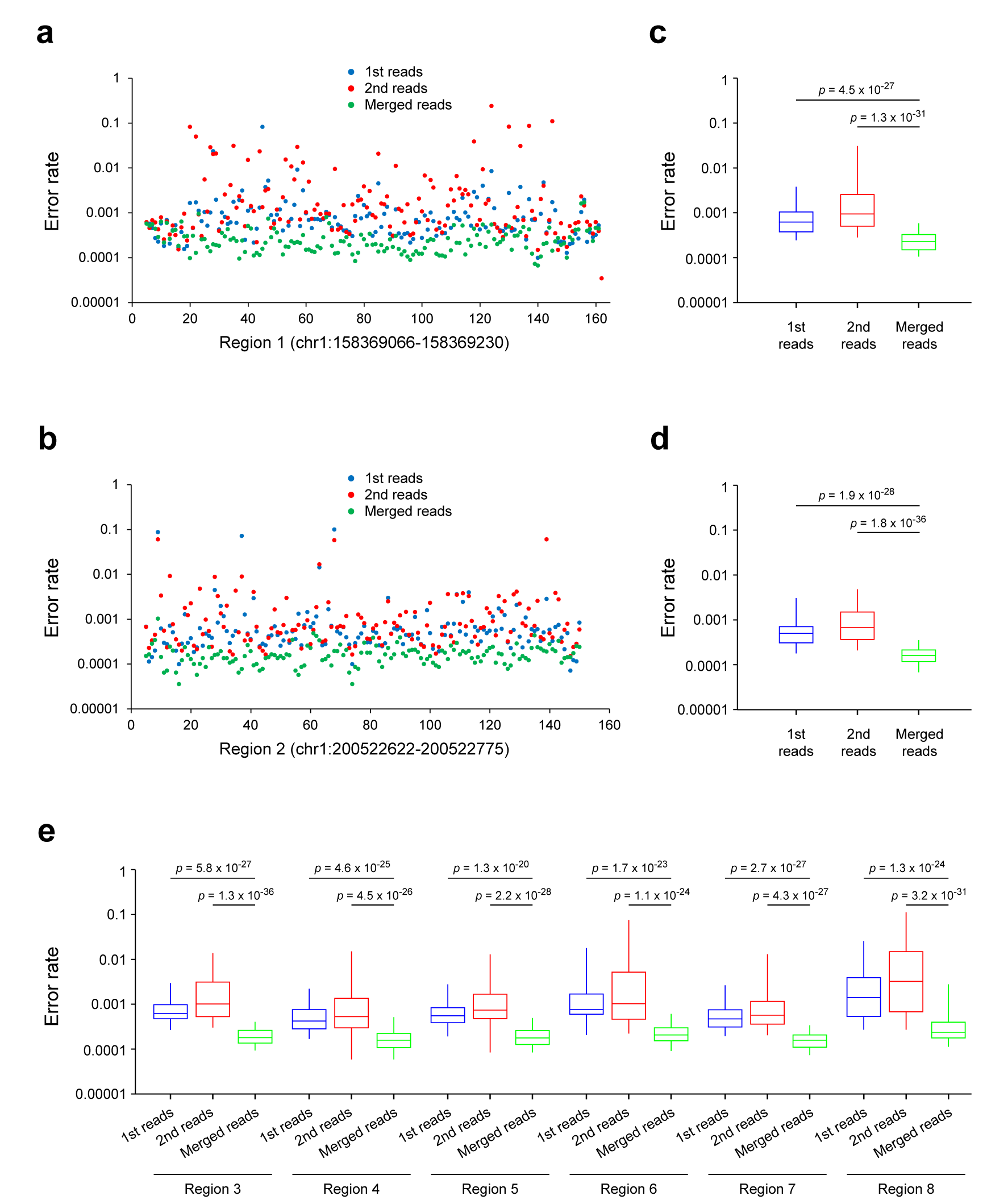

Supplement: S2 Fig — a,b. Error rates within the region where the first and second reads overlap for Region 1 (a) and 2 (b). Note that the merged reads consistently showed lower error rates than the first and second reads. c-e, Box plots showing error rates in Region 1 (c), 2(d) and 3–8 (e). 95th, 75th, 25th and 5th percentile and the median value are shown. The Mann–Whitney U test was performed. (TIF) [file pgen.1005932.s002.tif]
